# Corrosion-Induced Mass Loss Measurement under Strain Conditions through Gr/AgNW-Based, Fe-C Coated LPFG Sensors

**DOI:** 10.3390/s20061598

**Published:** 2020-03-13

**Authors:** Chuanrui Guo, Liang Fan, Genda Chen

**Affiliations:** Department of Civil, Architectural and Environmental Engineering, Missouri University of Science and Technology, Rolla, MO 65401, USA; cggfb@mst.edu (C.G.); lf7h2@mst.edu (L.F.)

**Keywords:** corrosion sensor, mass loss measurement, LPFG sensor, graphene, silver nanowire, electrochemical test, various strain level

## Abstract

In this study, graphene/silver nanowire (Gr/AgNW)-based, Fe-C coated long period fiber gratings (LPFG) sensors were tested up to 72 hours in 3.5 w.t% NaCl solution for corrosion-induced mass loss measurement under four strain levels: 0, 500, 1000 and 1500 µε. The crack and interfacial bonding behaviors of laminate Fe-C and Gr/AgNW layer structures were characterized using Scanning Electron Microscopy (SEM) and electrical resistance measurement. Both optical transmission spectra and electrical impedance spectroscopy (EIS) data were simultaneously measured from each sensor. Under increasing strains, transverse cracks appeared first and were followed by longitudinal cracks on the laminate layer structures. The spacing of transverse cracks and the length of longitudinal cracks were determined by the bond strength at the weak Fe-C and Gr/AgNW interface. During corrosion tests, the shift in resonant wavelength of the Fe-C coated LPFG sensors resulted from the effects of the Fe-C layer thinning and the NaCl solution penetration through cracks on the evanescent field surrounding the LPFG sensors. Compared with the zero-strained sensor, the strain-induced cracks on the laminate layer structures initially increased and then decreased the shift in resonant wavelength in two main stages of the Fe-C corrosion process. In each corrosion stage, the Fe-C mass loss was linearly related to the shift in resonant wavelength under zero strain and with the applied strain taken into account in general cases. The general correlation equation was validated at 700 and 1200 µε to a maximum error of 2.5% in comparison with 46.5% from the zero-strain correlation equation.

## 1. Introduction

Corrosion is one of the most concerning issues that cause the deterioration of steel rebar and steel members in civil infrastructure. To evaluate the remaining load capacity of aging structures, engineers must determine the mass loss of cross sections induced by corrosion under operational conditions. Conventional corrosion detection techniques mainly focused on the indirect measurement of moisture, humidity, corrosion-induced strain and stress, and electrochemical parameters such as open circuit potential (OCP) [1,2]. Recently, fiber optic sensors have also been developed for indirect corrosion detection due to its compact size, immunity to electromagnetic interference and robustness under harsh environments. For example, Fiber Bragg Grating (FBG) [3] and Brillouin Optical Time Domain Analysis (BOTDA) [4] were utilized to measure the corrosion induced strain on steel rebar. FBG sensor coated with a functional polymer layer was utilized for moisture measurement in concrete [5]. Even though these methods can measure the indirect parameters precisely, the correlation between these parameters and the mass loss of steel rebar or members remains unknown due to the complicated corrosion process and environment. Furthermore, such a correlation varies with different structures. It is a challenge, if not impossible, to develop a universal correlation for corrosion-induced mass loss assessment. Therefore, direct measurement of the corrosion-induced mass loss is necessary.

Several mass loss measurement techniques have been developed recently based on the use of long period fiber gratings (LPFG) sensors. A nano iron/silica and polyurethane coated LPFG was first developed in 2015 to monitor the corrosion-induced mass loss [6]. For improved robustness and accuracy, a thin silver film was deposited in 2016 on a LPFG surface and the coated LPFG was then electroplated to form a Fe-C layer outside the silver film [7]. The electroplated Fe-C layer had the same key chemical components ratio as the steel rebar to be monitored. Therefore, the corrosion process of the Fe-C layer was identical to the steel member or rebar when subjected to the same corrosion environment. Correlation between the resonant wavelength shift of the sensor and the mass loss of the Fe-C layer was established. Since the silver film had high reflectivity, less light energy was transmitted into the Fe-C layer. Therefore, the sensitivity and service life of the Fe-C electroplated sensor were limited. To address this issue, the silver film was replaced in 2019 by a graphene/silver nanowires (Gr/AgNW) composite—a transparent conductive film for Fe-C electroplating on the LPFG surface [8]. Compared to the silver-based sensor, the sensitivity and service life of the Gr/AgNW based sensor increased by over 90% and 110%, respectively.

When the Fe-C coated LPFG sensors are placed in proximity and bonded to steel elements in practical applications, they are subjected to strain considerations. How the strain-induced cracks on the Fe-C layer affect the corrosion mechanism of the Fe-C layers and the monitoring performance of the sensors remains unknown. The correlation between mass loss (η) and wavelength shift (Δλ) obtained at zero strain [8] may not be accurate. 

In this study, low pressure chemical vapor deposition (LPCVD) with methane (CH_4_) gas as a precursor is utilized for graphene production on a copper catalyst substrate. Silver nanowires are then doped onto the graphene film to form a Gr/AgNW composite, which is wet transferred and adhered to the surface of a LPFG sensor under negative pressure. Next, Fe-C is electroplated onto the Gr/AgNW coated LPFG corrosion sensor. Finally, the corrosion sensor is tested in 3.5 w.t% NaCl solution under four strain levels (0, 500, 1000 and 1500 µε) for 72 h. Mass loss percentages of the Fe-C layer and sensitivity of the sensor at each strain are obtained from the optical transmission spectra and electrochemical impedance spectroscopy. A modified η-Δλ correlation is then established from the four groups of corrosion test results under tension, taking into account the effect of strains. The modified correlation is validated from test data obtained under additional two strain levels (700 and 1200 µε). Its accuracy is compared with that at zero strain correlation.

## 2. Materials and Methods

### 2.1. Long Period Fiber Gratings (LPFG) Sensor

LPFG is a fiber optic device with the refractive index of its core modulated periodically in the order of hundred microns [9]. The fundamental mode in the fiber core will be coupled with various cladding modes due to the long period gratings and thus generate a series of resonant wavelengths in the transmission spectrum.

(1) $$\lambda_{res}=(n_{eff}^{co}-n_{eff}^{cl,0j})\Lambda$$

As indicated in Equation (1), the resonant wavelength $\lambda_{res}$ is proportional to the grating period Λ and the difference in effective refractive index between the core $n_{eff}^{co}$ and the *j*th cladding mode $n_{eff}^{cl,0j}$. The effective refractive index of the cladding is determined by the indices of the core, the cladding and the surrounding medium [9]. In comparison with Fiber Bragg Gratings (FBG) in sub-micron grating period, which couples the forward propagating mode to the backward counterpropagating mode within the fiber core, LPFG sensors have the unique capability of monitoring the change in ambient refractive index with extended applications in chemical, environmental, biological and other related fields [10,11,12,13,14].

**Figure 1:**
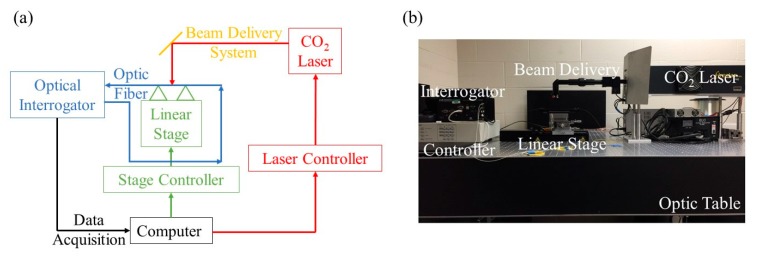
The CO_2_ laser grating system: (**a**) schematic view, and (**b**) laboratory setup.

In this study, a CO_2_ laser (Synrad Firestar V40) inscription method is utilized to fabricate LPFG sensors on bare optic fibers (Corning SMF 28e+, 125 µm in diameter) that are precleaned with isopropyl alcohol and then fixed on a linear stage for grating. As shown in Figure 1, the CO_2_ laser controlled by a computer generates and sends a laser beam to an optical fiber for point-by-point gratings. The beam delivery system contains a ZnSe cylindrical lens (Lasermech) that converts a round shape of the laser beam into an approximately 90 µm wide line shape to increase the resolution between gratings. The grating period (353 µm) is controlled by the motorized linear stage (Newport ILS100HA) with an accuracy of 0.1 µm, which is further controlled through the computer. The quality of the fabricated LPFG (approximately 4 cm in total length) is monitored by checking the transmission spectrum acquired through an optical interrogator (Micron Optics Si255), and fed back to the computer for any necessary adjustment in the fabrication process. The finished LPFG reveals a LP06 cladding mode at a resonant wavelength of 1550 nm.

### 2.2. Graphene/Silver Nanowires (Gr/AgNW) Transparent Electrode

In the previous study [15], the increased thickness of the silver layer from 0.8 µm to 1.2 µm reduced the sensitivity of the sensor by approximately 60%. This is because that the evanescent field surrounding the LPFG attenuates exponentially with the distance from the surface of optical fiber and the silver layer (higher reflective index) blocks more energy of the couple mode from reaching to the Fe-C layer. As a result, only the corrosion process of an inner portion of Fe-C layer will be detectable, compromising the service life of the sensor. Therefore, a transparent conductive film (TCF) is needed in the process of Fe-C electroplating to improve the sensitivity and service life of the LPFG sensor. In the past decades, TCF has received increasing attention in applications such as sensors, actuators, optical devices and touchscreens [16,17]. The most commonly used TCF in these industries was a Indium Tin Oxide (ITO) film, which is optically transparent in the visible light range and electrically conductive [18,19]. Even so, the ITO film still has the following drawbacks: (1) like all other TCFs, compromise must be made between the transparency and conductivity since a thicker ITO film will increase charge carriers but reduce its transparency, (2) the synthesis and deposition process of a thin-layer ITO requires the process of physical vapor deposition (PVD) such as sputtering, which is expensive and energy intensive, and (3) the ITO layer is too brittle to be used as a flexible film. Conductive polymer such as PEDOT: PSS was used as an alternative material for TCF [20]. It is less expensive, flexible and environmentally friendly. However, its conductivity is lower than the inorganic materials such as the ITO.

Graphene is a two-dimensional material consisted of single layer carbon atoms in hexagonal lattice. It was originally produced from graphite in laboratory through mechanical exfoliation [21,22,23]. Due to its excellent optical, electrical, mechanical and thermal properties, graphene was used as a TCF in many applications such as flexible touchscreen, organic light emitting diode (OLED), chemical sensor, soft robotics, actuators and biological devices [16,17,24,25,26]. In order to achieve industry-scale productions, various graphene synthesis techniques were developed by researchers [27,28,29,30]. The most effective technique was chemical vapor deposition (CVD) due to its robust production capability for large area monolayer graphene. It was based on the chemical decomposition of carbon precursors under high temperature and deposition of the carbon atoms on a metal catalyst surface such as copper (Cu) and nickel (Ni).

Even though the theoretical properties of graphene are fascinating, as-grown graphene on the metal catalyst (Cu, Ni) through the CVD process has intrinsic disorders at grain boundaries induced by the metal recrystallization under high temperature (>1000 °C) [31]. The atomic orientation of a polycrystalline graphene layer is discontinuous at the grain boundary, reducing its conductivity. Cracks and wrinkles are also introduced during the transfer of graphene layer in wet and dry conditions or through electrochemical bubbling [32], which will further reduce the electrical conductivity and mechanical strength of as-grown graphene.

To improve the conductivity and strength of the as-grown graphene, silver nanowires (AgNW) were doped onto the graphene film to form a Gr/AgNW composite [19,33]. The AgNW network connects the defects and wrinkles in the graphene layer and thus enhance the conductivity and mechanical strength. Optical transmittance of the composite is not compromised since the diameter of the AgNW is only 30 nm. Compared to the silver layer on the LPFG surface for electroplating, the Gr/AgNW nano composite is more transparent while maintaining comparable conductivity, which allows light to penetrate a thicker Fe-C layer from the coupled cladding mode and thus increases the sensitivity and service life of the sensor.

In this study, a 2 cm × 10 cm copper foil was first inserted into the quartz tube of a specially assembled LPCVD system, and then annealed 30 min at 1030 °C with a hydrogen flow at 5 sccm (standard cubic centimeter per minute). Another 5 sccm methane was then flown into the chamber for 5 min to grow a monolayer graphene on the copper surface. The Gr/Cu sample was cooled down to room temperature for next step transfer.

**Figure 2:**
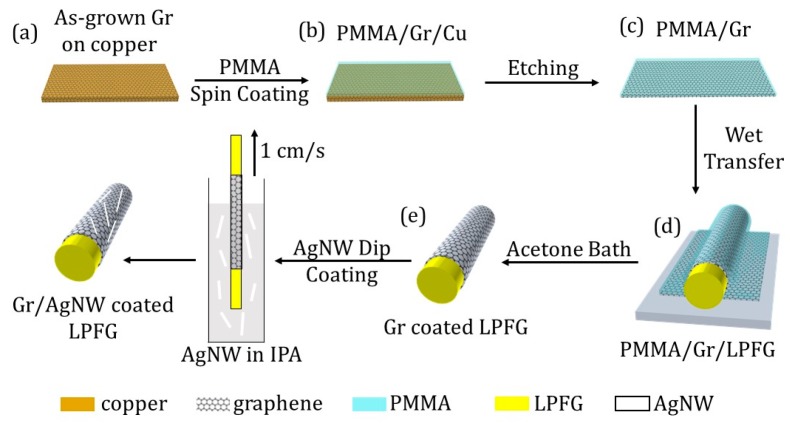
Gr/AgNW coating process on the LPFG sensor.

Figure 2 shows the transfer process of graphene from the flat copper surface to the curve surface of a LPFG sensor. As shown in Figure 2, the Gr/Cu sample was spin coated with 46 gm/mL PMMA-chlorobenzene solution at the speed of 4000 rpm. The sample was then heated at 180 °C to evaporate the chlorobenzene, leaving a thin PMMA film on the top. The copper foil was etched away by floating the PMMA/Gr/Cu on the copper etchant for 2 h. The PMMA/Gr film was cleaned in deionized water twice and then transferred onto the LPFG surface. An acetone bath was performed on the sample to dissolve the PMMA film and left the Gr coated LPFG. This process was repeated three times to create a three-layer graphene coated on the LPFG surface. Finally, 0.4 mg/mL AgNW in isopropyl alcohol (IPA) was dip coated on the sample with a speed of 1 cm/second to fabricate the Gr/AgNW coated LPFG.

### 2.3. Fe-C Electroplating on a Gr/AgNW Film of the LPFG Sensor

**Figure 3:**
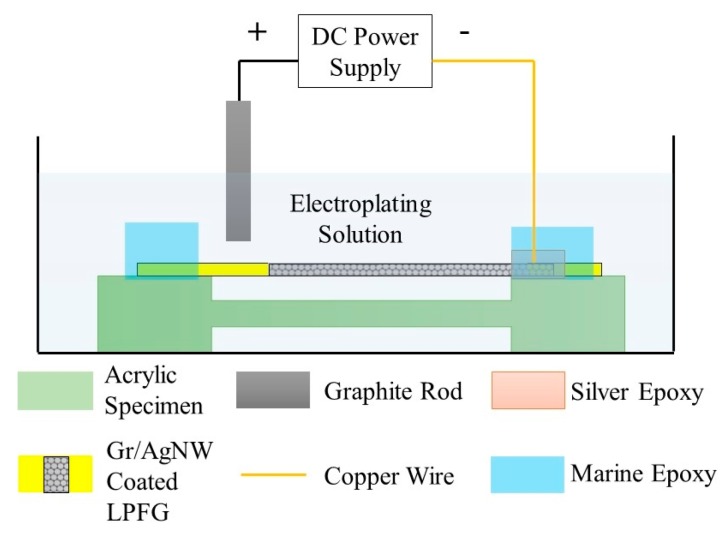
Schematic illustration of the Fe-C electroplating setup.

As shown in Figure 3, one end of the copper wire (0.3 mm in diameter) was bonded to the Gr/AgNW film of a representative sensor using conductive silver epoxy adhesive (MG Chemical 8331). The other end of the copper wire was connected to the negative electrode on the DC power supply. A graphite rod was connected to the positive electrode to form the circuit loop. A corrosion resistant marine epoxy (Loctite) was applied to cover and protect the copper wire-Gr/AgNW-silver epoxy bond and fix the sensor on the acrylic specimen. The sample was immersed in the electroplating solution (40 g/L FeSO_4_·7H_2_O, 3.0 g/L L-ascorbic acid and 1.2 g/L citric acid). The electroplating process was conducted under 5 mA current for 1.5 hours to form an approximately 30 µm thick Fe-C layer on the LPFG surface.

## 3. Experimental Setup

**Figure 4:**
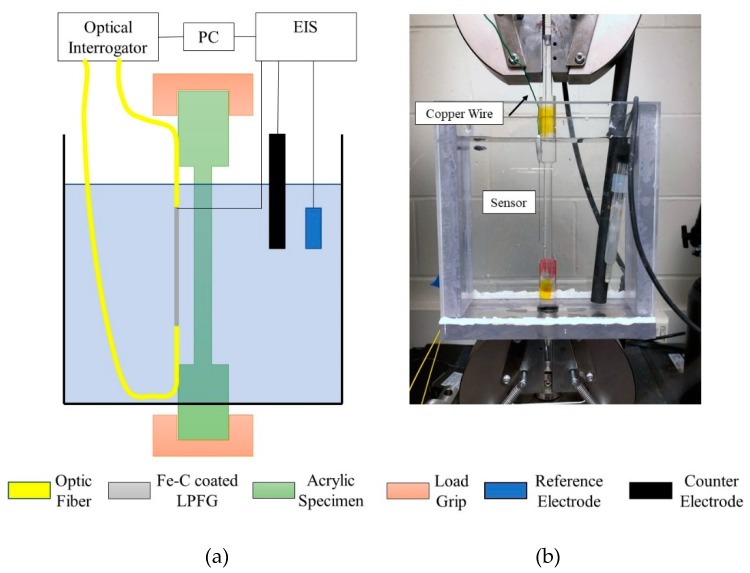
Tensile test setup of a Fe-C coated sensor in 3.5wt.% NaCl solution: (**a**) schematic view, and (**b**) laboratory test setup.

To investigate the corrosion monitoring performance of the proposed sensor under various strain levels, a 72-hour corrosion test with acquisition of optical transmission spectra and EIS measurement was conducted on a load frame (Instron 5965). As shown in Figure 4, the fabricated sensor was fixed on an acrylic dog-bone specimen first. The entire specimen was inserted into an acrylic container with a circular hole at the bottom and sealed by a rubber O-ring. The top and bottom of the specimen were griped by the Instron load frame (5 kN max force, 0.001 N resolution). The optic fiber loop was connected to a 1 kHz optical interrogator (Micron Optics Si255) to collect the transmission spectra every two hours. For EIS tests, the Fe-C layer was connected through the copper wire bonded on the Gr/AgNW film to the EIS equipment (Gamry Potentialstat/EIS 300) as the working electrode. The connection part was covered by marine epoxy to avoid contact with the electrolyte. The stabilized open circuit potential (OCP) was measured first. EIS measurements were then taken at 5 points per decade under a 10 mV sinusoidal potential around the OCP with an excitation frequency of 5 mHz to 100 kHz. The container was filled with 3.5 wt.% NaCl solution to fully immerse the sensor. Four strain levels: 0, 500, 1000 and 1500 µε were applied and sustained through the load frame. To make sure the strain on the sensor was accurate, another bare FBG was fixed on the acrylic specimen together with the corrosion sensor to obtain the strain readout and adjust the load accordingly. Three tests of each strain level were conducted for repeatability consideration.

## 4. Results and Discussion

### 4.1. Characterization of the Crack Distribution on Fe-C Layer

Distribution of the cracks on Fe-C layer under various strain levels was characterized first. The Fe-C coated LPFG was applied with three strained conditions (500, 1000 and 1500 µε) and fixed on an 18 mm diameter holder with 20 MPa glue to take the SEM images.

**Figure 5:**
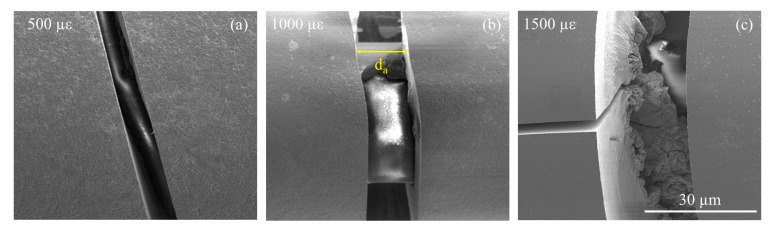
Transverse cracks on the Fe-C layer at (**a**) 500 µε, (**b**) 1000 µε, and (**c**) 1500 µε.

Figure 5 shows SEM images of the transverse cracks on a representative Fe-C layer at each strain level. The crack width *d_a_* increases from 7.9 to 24.9 µm as the applied strain increases from 500 to 1500 µε. To examine spacing of the transverse cracks, SEM images were taken from one crack and then scanned continuously to an adjacent crack. A full image covering two adjacent transverse cracks was then obtained by stitching the series of continuous images. Multiple stitched images were taken to obtain the average spacing of the representative Fe-C layer.

**Figure 6:**
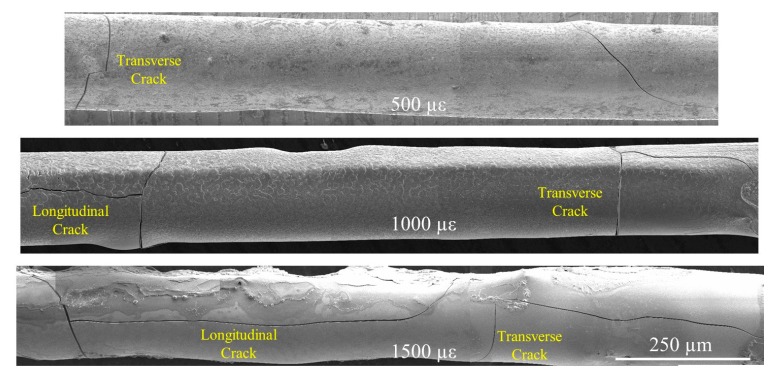
Stitched SEM images along axial direction of the fiber showing two adjacent transverse cracks at each strain.

Figure 6 shows the stitched SEM images of two adjacent transverse cracks from a representative sensor at each strain level. Note that a few transverse cracks appeared in Figure 6 are inclined, the center of which is referred to as their axial location when their spacing is calculated. It can be seen from Figure 6 that spacing of the transverse cracks slightly decreases with the increased strain level, which is mainly governed by the bond strength between the Fe-C layer and its underlying substrate. In addition, longitudinal cracks appear at 1000 and 1500 µε; those at 1500 µε propagate at a longer distance. This is likely because, as the applied strain increases, the transverse fracture initiates and is then followed by the longitudinal cracks due to uneven distribution of the bonding strength between the fiber and Fe-C layer. Since the total length of all cracks increases with the applied strain, providing paths for NaCl solution, the corrosion process of the Fe-C layer is accelerated at higher strains.

**Figure 7:**
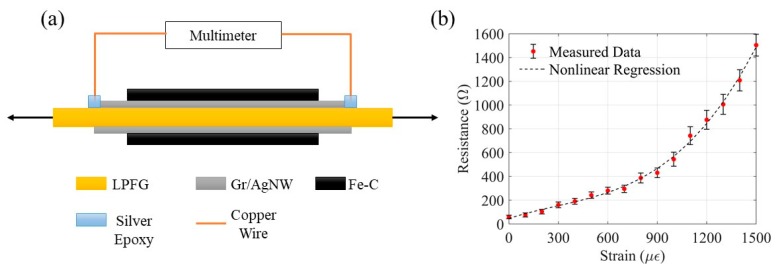
Sensor resistance measurement under various strain levels: (**a**) test setup, and (**b**) results.

To investigate the bonding integrity among the Fe-C layer, Gr/AgNW and optic fiber, the electrical resistances of three sensors were measured under various strain levels. As shown in Figure 7a, two pieces of copper wires were connected to the two ends of the Gr/AgNW layer via silver conductive epoxy and the multimeter for resistance measurement. Each sensor was fixed on a load frame and strained from 0 to 1500 µε with 100 µε interval. The resistance of the Gr/AgNW on the fiber is about 600 Ω from the previous work [8]. After electroplating, the Fe-C coated sensor has an initial resistance of 58.2 ± 15 Ω (average of three samples ± standard deviation). As shown in Figure 7b, including a regression cubic curve of the measured results, the resistance of the sensor with the Gr/AgNW and Fe-C layers in parallel increases cubically to 544 ± 59 Ω when strained from 0 to 1000 µε, which indicates that cracks emerged on the Fe-C layer and were extended at 1000 µε. When the applied strain exceeds 1000 µε, the resistance exceeds the 600 Ω threshold and increases at an accelerated pace. This is because the Fe-C layer was fully ruptured and the Gr/AgNW layer began to deform. The above resistance measurement indicates that the bond between the fiber and the Gr/AgNW film is stronger than that between the Gr/AgNW film and the Fe-C layer.

**Figure 8:**
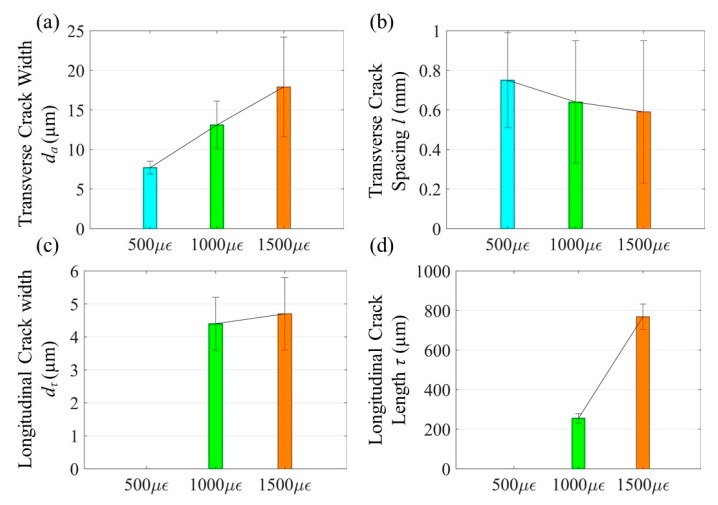
Strain dependence of: transverse crack (**a**) width and (**b**) spacing, longitudinal crack (**c**) width and (**d**) length.

Let *l* be the spacing of transverse cracks, dτ and τ the width and length of each longitudinal crack. Figure 8 summarizes the mean value and standard variation of each parameter from three tested samples at each strain. On one hand, the mean width and standard deviation of transverse cracks both increase linearly with the applied strain. The mean spacing and standard deviation of transverse cracks only change slightly with the applied strain likely due to the appearance and propagation of longitudinal cracks. Note that the variation in spacing of transverse cracks even at 500 µε is high, compared to other parameters. On the other hand, while the mean width and standard deviation of longitudinal cracks change little, the mean length of longitudinal cracks increases dramatically with the applied strain.

### 4.2. Transmission Spectra of the Corrosion Sensors

**Figure 9:**
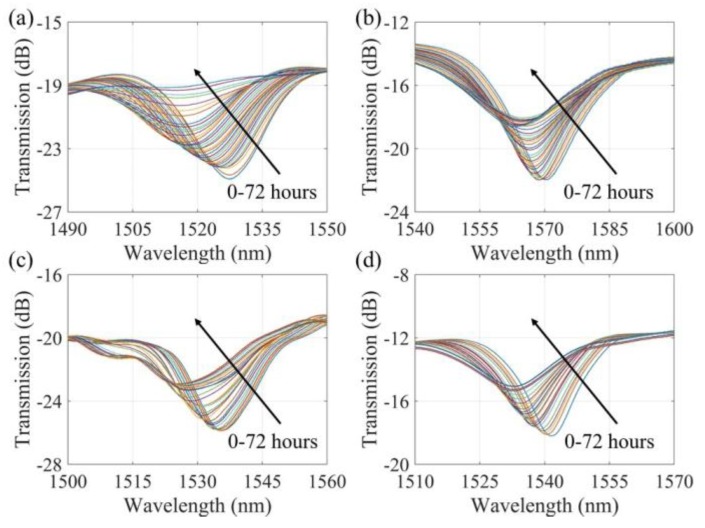
LPFG transmission spectra under various strains: (**a**) 0, (**b**) 500, (**c**) 1000 and (**d**) 1500 µε.

**Figure 10:**
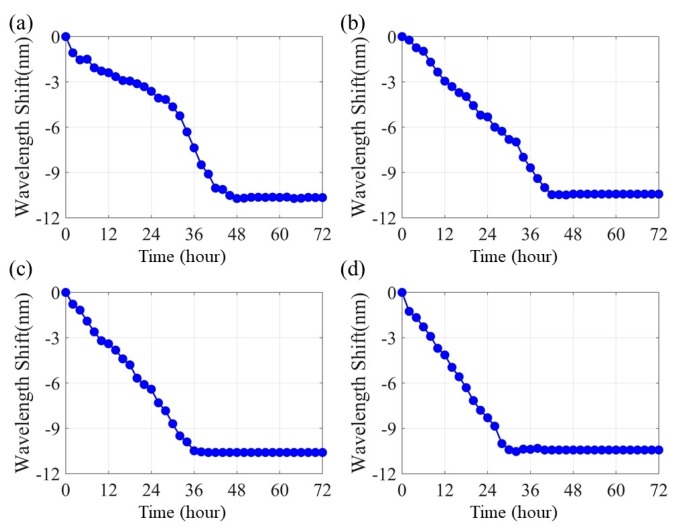
Resonant wavelength shift over time: (**a**) 0, (**b**) 500, (**c**) 1000 and (**d**) 1500 µε.

Figure 9 shows the transmission spectra shift of each sensor. The pitting corrosion, electrolyte penetration and Fe-C layer corrosion will increase the effective refractive index of the surrounding medium of the LPFG sensor and thus the resonance wavelength will have a trend of blue shift. On the other hand, since the accumulated corrosion product blocked more light energy from the evanescent field, the energy attenuation at the resonance wavelength will be reduced and thus the transmission spectra became broad and shallow. Resonant wavelengths were extracted from the spectra and presented in Figure 10 as a function of immersion time. At zero strain, the resonant wavelength ceased to change at about 48 h, which represents the operation time of the sensor. As the applied strain increased to 500, 1000 and 1500 µε, the operation time of the sensor was reduced to 42, 36 and 28 h, respectively. This is because the strain-induced cracks in the Fe-C layer accelerated the penetration of NaCl solution through the Fe-C layer, and thus corrosion rate of the wet Fe-C in contact with the solution. On the other hand, the total shifts in resonant wavelength of all four strain cases remained nearly the same, which is 10.4 nm. The total resonant wavelength shift is thus independent of the level of applied strains.

### 4.3. Electrochemical Impedance Spectroscopy (EIS)

**Figure 11:**
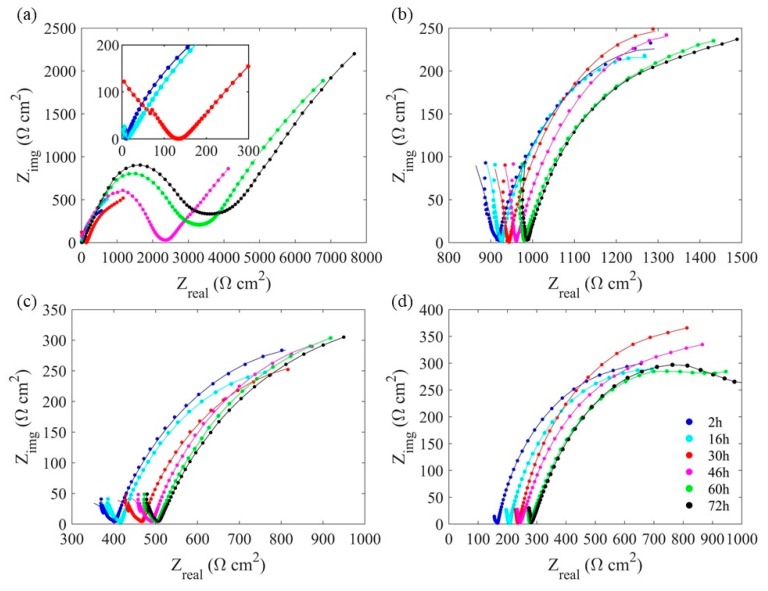
Change of Nyquist plots of the Fe-C layer under (**a**) 0, (**b**) 500, (**c**) 1000 and (**d**) 1500 µε in 3.5 wt. % NaCl solution up to 72 h.

Figure 11 shows the Nyquist plots of four Fe-C coated LPFG sensors at different strain levels from the EIS tests. Each plot includes several curves similar in pattern, each with two semicircles. The small semicircle in diameter, on the left side, implies the pore resistance of the combined Fe-C layer and Gr/AgNW film. The large semicircle indicates the charge transfer resistance at the interface between the Fe-C layer and its surrounding electrolyte [8,15]. Compared with Figure 11a, the diameters of the small semicircles in Figure 11b–d show a decreasing trend with the increase of the applied strain because the strain causes cracks in the Fe-C layer, as shown in Figure 5, and allows more electrolyte to arrive at the interface between the Fe-C layer and Gr-NW, resulting a reduced resistance. The diameters of the large semicircles are also reduced since the cracks permit more electrolyte in contact with the Fe-C layer, accelerating the Fe-C corrosion process. In addition, both the charge transfer resistance and the pore resistance increase over time due to thinning of the Fe-C layer and the gradual formation of a thick corrosion-product layer such as ferrous oxides.

**Figure 12:**
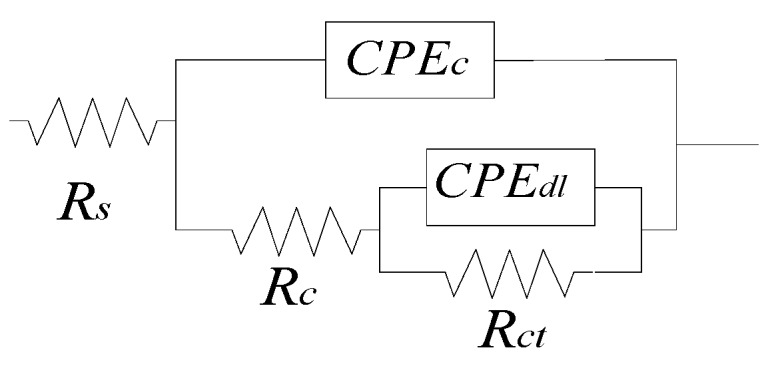
An equivalent electrical circuit (EEC) model.

An equivalent electrical circuit (EEC) model as shown in Figure 12 are used to fit the EIS test data. In Figure 11, the dotted points and solid lines denote experimental data and fitting curves with the EEC model, respectively. Specifically, *R_s_* represents the solution resistance; *CPE_c_* and *R_c_* represent capacitive behavior and pore resistance of the combined Fe-C and Gr/AgNW film, respectively; and *CPE_dl_* and *R_ct_* represent the double layer capacitance and the charge transfer resistance at the interface between Fe-C layer and the surrounding electrolyte [34,35]. In this study, a constant phase element (*CPE*) was used to replace a pure capacitor due to the irregularity of the Fe-C layer, the inherent defects in Gr/NW film, the random distribution of Ag nanowires, and the random distribution of micro-corrosion cell [15]. A *CPE* is defined by two parameters Y and n, and its impedance is represented by:

(2) $$Z_{CPE}=Y^{-1}{\left(j\omega \right)}^{-n}$$

where Y is a parameter with dimension of Ω-sec^n^/cm^2^, which is proportional to the pure capacitance, ω is the angular frequency in rad s^−1^, and n is an index that represents the deviation from a pure capacitor [34,35].

**Figure 13:**
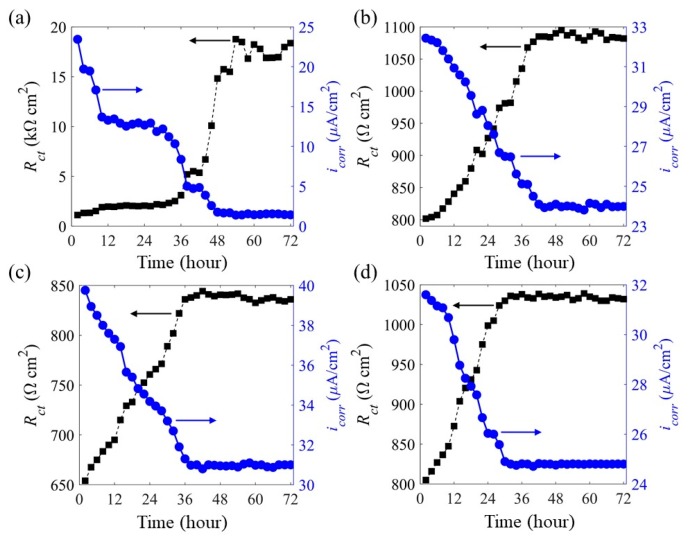
Charge transfer resistance and corrosion current density of the Fe-C layer at (**a**) 0, (**b**) 500, (**c**) 1000 and (**d**) 1500 µε.

**Figure 14:**
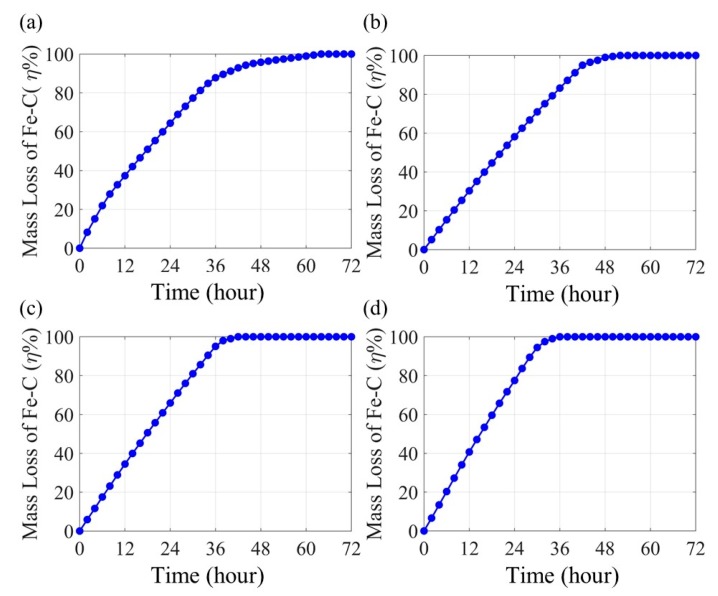
Fe-C mass loss over time at (**a**) 0, (**b**) 500, (**c**) 1000 and (**d**) 1500 µε.

Figure 13 shows the charge transfer resistance and associated corrosion current density of the Fe-C layer at each strain. The corrosion current density was calculated based on the entire surface area over the LPFG sensor. Compared to the zero-strain result in Figure 13a, the charge transfer resistance of the strained condition in Figure 13b–d increased linearly over time. Since the charge transfer resistance (*R_ct_*) is inversely proportional to the corrosion current density (*i_corr_*) based on the Stern–Geary equation *i_corr_* = B/*R_ct_* (B = 26 mV) [7,36], it can be related to the mass loss of the Fe-C layer through the Faraday Law. The 100% mass loss of the Fe-C layer was determined by the trend of *Rct* and corrosion current density *i_corr_*. When *R_ct_* and *i_corr_* were stable, the Fe-C layer was defined as 100% corroded. Compared with the non-strained sensor, the strained sensors had more cracks on the Fe-C surface, which creates paths for the electrolyte to get in direct contact with the Gr/AgNW layer. As a result, the corrosion current density was determined by the gradually corroded Fe-C layer and the Gr/AgNW layer together, which led to a relatively smaller change of the *i_corr_*. Furthermore, since the NaCl solution was in contact with the Gr/AgNW layer all the time, the corrosion current density will not decrease to zero, compared to the non-strained sensor. For the non-strained sensor, the corrosion product accumulated on the Fe-C surface [8] to make the *R_ct_* increased to 20 kΩ/cm^2^. However, for the strained sensors, the NaCl solution was in direct contact with the Gr/AgNW layer through the cracks on the Fe-C layer, *R_ct_* was dramatically reduced, compared to the non-strained sensor. The accumulated mass loss over time was normalized by the initial Fe-C mass and presented in Figure 14. It can be seen from Figure 14 that the accumulated mass loss of the Fe-C under strained conditions increased almost linearly over time and reached 100% in a shorter time than at zero strain. Under strain conditions, the Fe-C layer was thus corroded away more rapidly with an increasing average corrosion rate. It experienced both pitting corrosion at crack locations and uniform corrosion on the surface of the Fe-C layer. However, the NaCl solution directly contacted the Gr/AgNW layer at the crack locations, the charge transfer resistance was determined by the gradually corroded Fe-C layer and the stable Gr/AgNW layer together. As a result, the measured initial corrosion rate of the Fe-C layer in approximately the first 8 hours was even slower than that due to uniform corrosion on its entire surface at zero strain. Without notable cracks, the Fe-C layer was corroded as the NaCl solution wetted various surface dents and then penetrated through the Fe-C layer gradually.

### 4.4. Correlations between the Mass Loss and the Resonant Wavelength Shift

**Figure 15:**
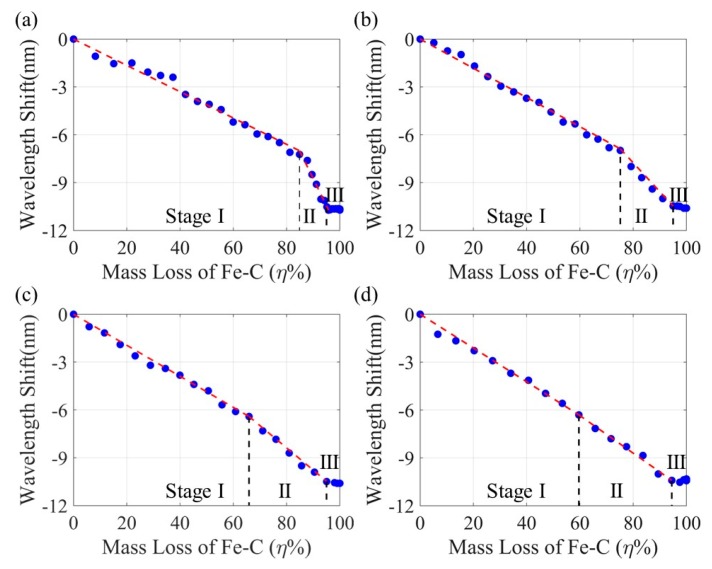
Resonant wavelength shift with Fe-C mass loss at (**a**) 0, (**b**) 500, (**c**) 1000 and (**d**) 1500 µε.

By combining Figure 9 and Figure 14, the shift in resonant wavelength of the Fe-C coated LPFG sensor can be correlated to the Fe-C mass loss as presented in Figure 15. Each correlation curve can be divided into three stages [8] with low, high and zero wavelength sensitivities to the mass loss, respectively. Stages I and II are dominated by the effect of Fe-C layer thinning and NaCl solution saturation on the evanescent field in the proximity of the LPFG sensor. The saturation effect likely includes two parts: liquid coverage along the length of the LPFG sensor and change in its refractive index from the surrounding Fe-C layer to the NaCl solution [37]. Stage III represents a near completion of corrosion process in the Fe-C layer and the LPFG sensor becomes fully submerged in the NaCl solution. At zero strain, uniform corrosion occurred on the surface of the Fe-C layer in Stage I until locally breached. Once the Fe-C layer was fully penetrated, NaCl solution reached the surface of the LPFG sensor both perpendicularly at the penetration points and laterally along the weak interface between the optical fiber and the Gr/AgNW film in Stage II. Under strained conditions, the NaCl solution penetrated the Fe-C layer locally through the strain-induced cracks from the beginning of corrosion process, diminishing the importance of Fe-C layer thinning. The larger number and slightly smaller spacing of transverse cracks as shown in Figure 8b led to the less effect of uniform corrosion and thus the reduced mass loss at the end of Stage I.

It can be clearly seen from Figure 15 that, as the applied strain on the Fe-C coated LPFG sensor increased, the wavelength shift sensitivity to the mass loss increased in Stage I and decreased in Stage II. The interception between the two stages depended on the relative effects of Fe-C thinning and NaCl saturation. For practical applications, the mass loss (η) of the Fe-C layer is related to the directly measured wavelength shift (Δλ), based on the regression analysis of test data from three samples at each strain. The coefficient of correlations obtained in all cases exceed 0.9, indicating a great η-Δλ correlation. The mean ± standard variation of the mass loss can be estimated by Equation (3) in Stage I and Equation (4) in Stage II:

(3) $$\begin{array}{lll} \eta =(-10.99\pm 0.2)\Delta \lambda , & R^{2}=0.91 & 0\;\mu \varepsilon \\ \eta =(-10.31\pm 0.3)\Delta \lambda , & R^{2}=0.97 & 500\;\mu \varepsilon \\ \eta =(-9.52\pm 0.1)\Delta \lambda , & R^{2}=0.95 & 1000\;\mu \varepsilon \\ \eta =(-8.93\pm 0.1)\Delta \lambda , & R^{2}=0.94 & 1500\;\mu \varepsilon \end{array}$$

(4) $$\begin{array}{lll} \eta =(-2.89\pm 0.4)\Delta \lambda +62.25\pm 4.1, & R^{2}=0.93 & 0\;\mu \varepsilon \\ \eta =(-5.52\pm 0.2)\Delta \lambda +41.49\pm 3.7, & R^{2}=0.95 & 500\;\mu \varepsilon \\ \eta =(-7.14\pm 0.3)\Delta \lambda +22.93\pm 1.8, & R^{2}=0.92 & 1000\;\mu \varepsilon \\ \eta =(-8.47\pm 0.4)\Delta \lambda +4.96\pm 0.5, & R^{2}=0.96 & 1500\;\mu \varepsilon \end{array}$$

### 4.5. Modified Correlation Between the Mass Loss and Resonant Wavelength Shift

**Figure 16:**
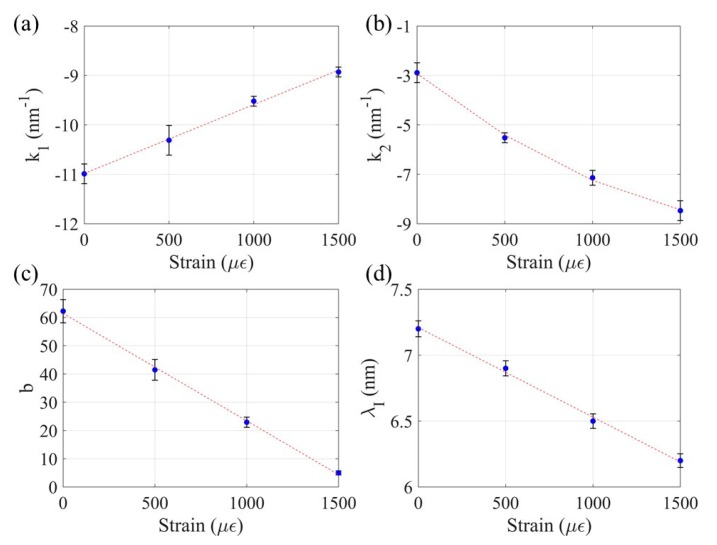
Curve fitting of (**a**) Stage I sensitivity, (**b**) Stage II sensitivity, (**c**) Intercept mass loss in Stage II correlation, and (**d**) Wavelength shift at the end of Stage I.

The four expressions in Equation (3) or Equation (4) can be combined into one general expression with the strain effect taken into account in various coefficients. The general η-Δλ correlation can thus be expressed into:

(5) $$\begin{array}{lll} \eta =k_{1}\Delta \lambda & \Delta \lambda \leq \lambda_{I} & \mathrm{Stage}\;I\\ \eta =k_{2}\Delta \lambda +b & \Delta \lambda >\lambda_{I} & \mathrm{Stage}\;\mathrm{II} \end{array}$$

where k_1_ and k_2_ are the mass loss sensitivity coefficients in Stage I and II, respectively, λ_I_ is the total resonant wavelength shift at the end of Stage I, and b is the interception mass loss of Stage II correlation. In Equation (5), η represents the mass loss in percentage. These four parameters were obtained from each strain case; their mean values were then curve fitted with the strain ε, as shown in Figure 16.

The curve fitting functions of the four parameters in Equation (5) are listed in Equation (6):

(6) $$\begin{array}{ll} k_{1}=0.0014\varepsilon -10.99 & {\;R}^{2}=0.93\\ k_{2}=1.3\times 10^{-6}\varepsilon^{2}-0.0056\varepsilon -3.93 & {\;R}^{2}=0.95\\ b=-0.0381\varepsilon +61.46 & {\;R}^{2}=0.91\\ \lambda_{I}=-6.8\times 10^{-4}\varepsilon +7.21 & {\;R}^{2}=0.96 \end{array}$$

Here, the coefficients of correlation of the four parameters with the applied strain all exceed 0.9, indicating satisfactory correlation curves. When the strain applied on the Fe-C coated LPFG sensor is known (e.g., measured), the four parameters (k_1_, k_2_, b and λ_I_) can be calculated from Equation (6). The general η-Δλ correlation in Equation (5) can then be applied in practice to estimate the mass loss from the shift in resonant wavelength extracted from the directly measured transmission spectra.

To validate the general correlation, two additional Fe-C coated LPFG sensors were fabricated and tested under 700 and 1200 µε in tension, respectively, in the container filled with 3.5 w.t.% NaCl solution using the same setup as shown in Figure 4. The mass loss and resonant wavelength were measured and correlated through the same procedure in previous work. The shift in measured resonant wavelength was also used in Equations (5, 6) to determine the estimated mass loss. 

Let η_m_ be the measured mass loss, η_c1_ be the mass loss calculated using the zero-strain η-Δλ correlation or the first expression in Equation (4), and η_c2_ be the mass loss calculated from the general η-Δλ expressions in Equation (5). The calculated mass losses were compared with the measured one in Table 1 to evaluate the error of different equations used. As shown in Table 1, the maximum error in mass loss estimation from the zero-strain correlation is 36.2% at 700 µε and 46.5% at 1200 µε. By using the general expressions in Equation (5), the maximum error in mass loss estimation is substantially reduced to 2.2% at 700 µε and 2.5% at 1200 µε.

## 5. Conclusions

In this study, Fe-C coated LPFG corrosion sensors were tested under four strain conditions in a container filled with 3.5w.t.%NaCl solution. The Fe-C mass loss was calibrated with the resonant wavelength shift of the LPFG sensor, taking strain effect into account. The calibrated correlation was compared with that at zero strain and applied to the cases under different strains. Based on extensive test data and analysis, the following conclusions can be drawn:

1. Under tensile loads, transverse cracks appear first on the Fe-C layer of LPFG sensors and are followed by the emerging of longitudinal cracks. As the applied strain increases from 500 to 1500 µε, the mean width of transverse cracks on three Fe-C layers increases linearly from 7.7 to 17.9 µm, and the mean spacing between the transverse cracks decreased from 0.75 to 0.59 mm. As the applied strain increases from 1000 to 1500 µε, the mean width of longitudinal cracks on the three Fe-C layers increases slightly from 4.4 to 4.7 µm, and the mean length of the longitudinal cracks increases dramatically from 255 to 770 µm. The spacing of transverse cracks and the length of longitudinal cracks are likely determined by the bond strength at the weak interface between the optical fiber and the laminate Fe-C and Gr/AgNW layer structure.
2. The correlation curve between the shift in resonant wavelength of a Fe-C coated LPFG sensor and the Fe-C mass loss can be divided into three stages with low, high and zero wavelength sensitivities to the mass loss, respectively. Stages I and II are dominated by the effect of Fe-C layer thinning and NaCl solution saturation on the evanescent field in the proximity of the LPFG sensor. Stage III represents a near completion of corrosion process in the Fe-C layer and the LPFG sensor becomes fully submerged in the NaCl solution. At zero strain, uniform corrosion occurs on the surface of the Fe-C layer in Stage I until locally breached. Once the Fe-C layer is fully penetrated, NaCl solution reaches the surface of the LPFG sensor both perpendicularly at the penetration points and laterally along the weak interface between the optical fiber and the Gr/AgNW film in Stage II. Under strained conditions, the NaCl solution penetrates the Fe-C layer locally through the strain-induced cracks from the beginning of corrosion process.
3. For practical applications, the Fe-C mass loss is related to the shift in resonant wavelength of the Fe-C coated LPFG sensor under 0, 500, 1000 and 1500 µε strain conditions. The mean mass loss sensitivity to the shift in resonant wavelength from three test samples decreases linearly from 10.99 nm^−1^ at zero strain to 8.93 nm^−1^ at 1500 µε in Stage I, and increase almost linearly from 2.89 nm^−1^ at zero strain to 8.47 nm^−1^ at 1500 µε in Stage II. The specific correlation equation at zero strain and the general correlation equation taking strain effect into account are compared and validated at 700 and 1200 µε, which represent two application cases in practice. The maximum error in mass loss estimation from the zero-strain correlation is 36.2% at 700 µε and 46.5% at 1200 µε. By using the general correlation equation, the maximum error in mass loss estimation is reduced to 2.2% at 700 µε and 2.5% at 1200 µε.

## Figures and Tables

**Table 1:** Comparison of the measured and calculated mass losses (%).

Δλ (nm)	η_c1_	700 µε				1200 µε			
		η _m_	Error	η_c2_	Error	η_m_	Error	η_c2_	Error
2	27.1	19.9	36.2%	20.2	1.3%	18.5	46.5%	18.1	1.9%
4	49.1	39.5	24.3%	40.5	2.2%	37.2	32.0%	37.9	1.7%
6	71.0	59.8	18.7%	60.6	1.4%	55.2	28.6%	56.7	2.5%
8	93.1	80.1	16.2%	78.8	1.2%	73.4	26.8%	75.2	2.2%
